# Neurotrauma admission disparities in Scotland: a 6-year analysis of socioeconomic and demographic trends

**DOI:** 10.3389/fneur.2026.1824343

**Published:** 2026-05-19

**Authors:** Andreas K. Demetriades, Wilco Peul

**Affiliations:** 1 Scottish Acquired Brain Injury Network (SABIN), NHS Scotland, Edinburgh, United Kingdom; 2 Department of Neurosurgery, Edinburgh Spinal Surgery Outcome Studies Group, Royal Infirmary Edinburgh, NHS Lothian, Edinburgh, United Kingdom; 3 Department of Neurosurgery, Leiden University Medical Center, Leiden, Netherlands; 4 Department of Neurosurgery, Haaglanden Medical Center, The Hague, Netherlands

**Keywords:** concomitant craniospinal trauma, demographic, disparities, neurotrauma, SIMD (Scottish Index of multiple deprivation), socioeconomic, spinal trauma, traumatic brain injury (TBI)

## Abstract

**Background:**

Neurotrauma, encompassing traumatic brain injury (TBI) and spinal trauma, remains a significant global cause of mortality and morbidity. Socioeconomic deprivation is associated with higher injury incidence and poorer outcomes, though gradients vary across healthcare systems. This study examines neurotrauma admission disparities across socioeconomic and demographic groups in Scotland over a 6-year period.

**Methods:**

A national population-based analysis of neurotrauma admissions in Scotland from 2014 to 2020 was conducted using data from Public Health Scotland. Admissions were categorized by sex, age group, injury severity (minor, moderate, major), and socioeconomic status using the Scottish Index of Multiple Deprivation (SIMD), where Quintile 1 represents the most deprived and Quintile 5 the least deprived areas. Year-on-year incidence trends, demographic patterns, and length of stay (LOS) were analyzed.

**Results:**

Total neurotrauma admissions increased from 1,098 in 2014 to 2,428 in 2020. A consistent socioeconomic gradient was observed across all injury severities: the most deprived group (SIMD 1) accounted for the highest proportion of admissions annually, while the least deprived (SIMD 5) had the lowest. Males demonstrated higher admission numbers than females across all severity levels. Age-related patterns revealed patients aged <16 years had the highest proportion of head injuries (73.0%), whereas those aged 16–44 and 45–54 had higher proportions of spinal injuries (48.9 and 48.4% respectively). Longer hospital stays were associated with greater deprivation, even after adjusting for age and injury severity.

**Conclusions:**

Substantial and persistent socioeconomic gradients exist in neurotrauma admissions across Scotland, with the most deprived communities bearing the greatest burden. These disparities persist despite universal healthcare coverage, suggesting deprivation influences injury exposure, resilience, and recovery pathways. Findings support targeted prevention strategies for high-risk groups and resource allocation informed by area-based deprivation indices to address neurotrauma as both a clinical and public health challenge.

## Introduction

Neurotrauma, including traumatic brain injury (TBI) and spinal trauma, remains a major cause of mortality, morbidity, and long-term disability worldwide. Global Burden of Disease analyses have shown that traumatic brain and spinal injuries continue to impose a substantial health burden across age groups and healthcare systems (1). Socioeconomic deprivation has repeatedly been associated with higher injury incidence, greater injury severity, and poorer post-injury trajectories, although the magnitude and pattern of these inequalities vary between countries and healthcare settings. Scotland provides an important setting in which to examine these relationships. Healthcare is delivered within a universal publicly funded system, yet substantial health inequalities persist. Trauma care in Scotland has undergone progressive national organization through the Scottish Trauma Network, which works across prevention, pre-hospital care, acute care, rehabilitation, and major incident planning, with regional trauma networks and major trauma centers supporting delivery of care across the country (2, 3). In parallel, service-mapping work from the Scottish Acquired Brain Injury Network (SABIN) has highlighted the complexity of neurotrauma pathways in Scotland, including variation in local service configuration, rehabilitation provision, and onward care arrangements (4).

In Scotland, socioeconomic context is commonly assessed using the Scottish Index of Multiple Deprivation (SIMD), an area-based relative measure derived from seven domains: income, employment, education, health, access to services, crime, and housing (5). SIMD can be grouped into quintiles, with Quintile 1 representing the most deprived areas and Quintile 5 the least deprived. Because these quintiles represent approximately equal population shares, they provide a practical framework for examining whether particular groups are over- or under-represented among hospital admissions (5).

Previous Scottish work has demonstrated sociodemographic gradients in trauma incidence and in the organization of brain injury services, but contemporary population-level analyses specifically examining neurotrauma admissions across socioeconomic and demographic groups remain limited (4, 6). This study therefore analyzed national neurotrauma admissions in Scotland from 2014 to 2020, focusing on temporal trends, demographic characteristics, and socioeconomic gradients by SIMD quintile. We hypothesized that neurotrauma admissions would be disproportionately concentrated in more deprived communities, despite delivery of care within a universal healthcare system.

## Methods

We conducted a national population-based observational analysis of neurotrauma admissions in Scotland between 2014 and 2020 using a Scottish neurotrauma dataset derived from Public Health Scotland administrative data. Admissions were classified according to patient sex, age group, injury type, injury severity, and socioeconomic status. Socioeconomic status was assessed using the Scottish Index of Multiple Deprivation (SIMD), an area-based measure of relative deprivation in Scotland. SIMD ranks small areas according to multiple deprivation and can be grouped into quintiles, with Quintile 1 representing the most deprived areas and Quintile 5 the least deprived (5). Because SIMD quintiles represent approximately equal shares of the population, differences in admission proportions across quintiles may be interpreted as relative over-representation or under-representation of particular socioeconomic groups within the admitted population, while recognizing that SIMD is an area-level rather than an individual-level measure (5). Injury severity was classified as minor, moderate, or major for both head and spinal injuries according to the Injury Severity Score (ISS), as provided within the dataset. We analyzed annual admission counts, demographic patterns, and hospital length of stay (LOS) across these categories. Given the nature of the available administrative data, the analysis was descriptive and exploratory, and was designed to identify population-level patterns rather than infer causality.

## Results

Annual recorded neurotrauma admissions were examined across the study period overall and then stratified by SIMD quintile, sex, age group, injury type, and injury severity in order to describe temporal, demographic, and socioeconomic patterns within the national dataset.

**Figure 1:**
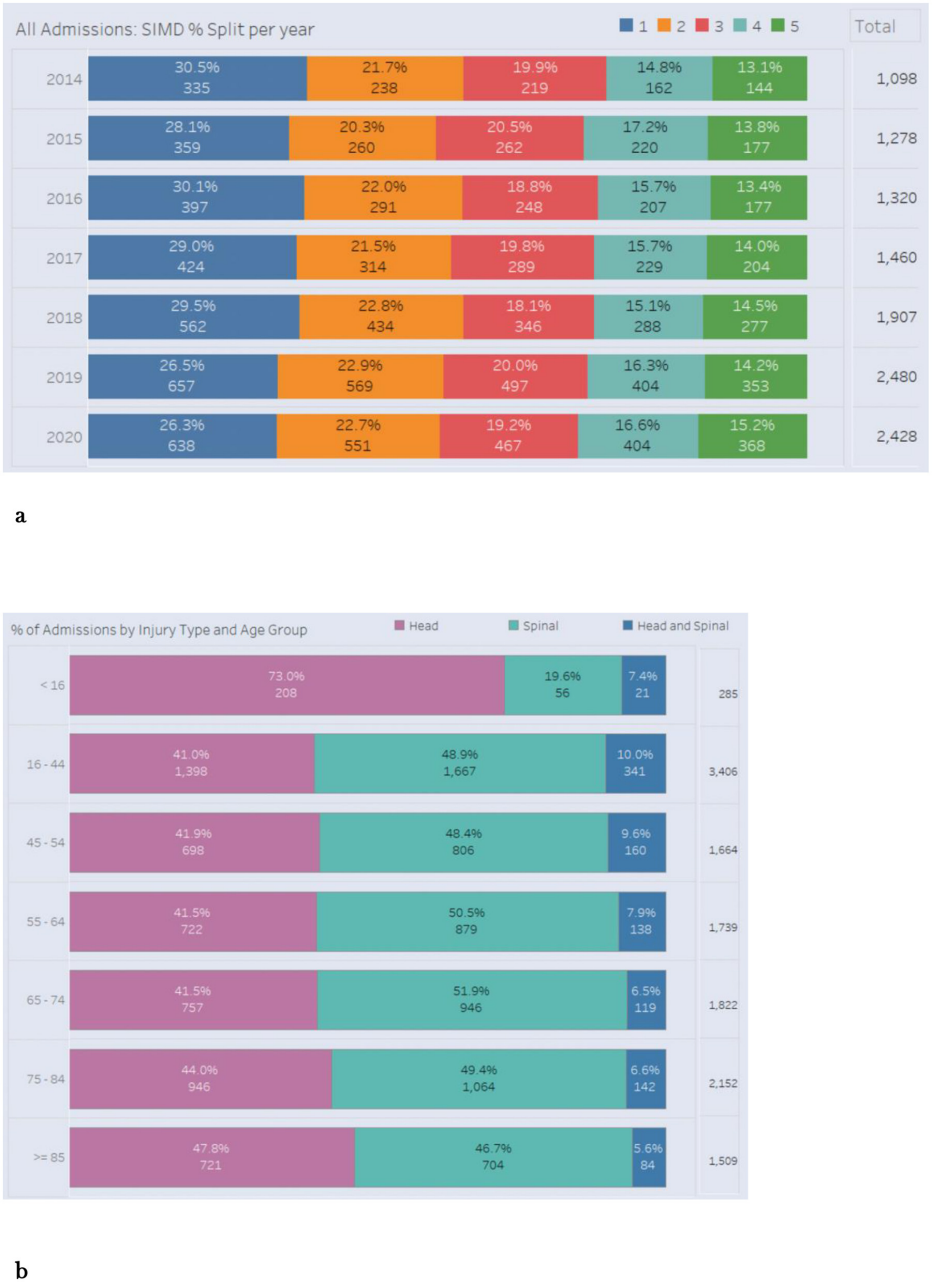
**(A)** All neurotrauma admissions 2014–2020, showing the SIMD split per year. SIMD 1 is the most deprived, and SIMD 5 is the least deprived. **(B)** Percentage split of neurotrauma admissions by Injury type (head injury; spinal injury; concomitant brain and spine injury) across age groups.

Recorded neurotrauma admissions in the dataset increased from 1,098 in 2014 to 2,428 in 2020. A consistent and pronounced socioeconomic gradient was observed: the most deprived group (SIMD 1) consistently accounted for the highest percentage and absolute number of admissions annually, while the least deprived group (SIMD 5) had the lowest. Because SIMD quintiles represent approximately equal population shares, this pattern indicates relative over-representation of individuals living in the most deprived areas and relative under-representation of those living in the least deprived areas among neurotrauma admissions. This trend was maintained across all severities of injury. Figure 1A details annual admissions by SIMD quintile. The increase over time should be interpreted as an increase in recorded admissions within the dataset rather than definitive evidence of a true increase in the underlying population incidence of neurotrauma.

Across both sexes, raw admission numbers increased over the study period. Males consistently demonstrated a higher number of admissions than females in all injury severity levels (minor, moderate, and major head and spinal injuries).

Specific age-related patterns emerged. Patients aged <16 years had the highest proportion of head injuries (73.0%), whereas those aged 16–44 and 45–54 had higher proportions of spinal injuries (48.9 and 48.4% respectively; Figure 1B). Length of hospital stay (LOS) data suggested longer stays were influenced by injury severity, age, comorbidities and socioeconomic status. Patients from more deprived SIMD categories had a longer LOS than the least deprived category even after adjustment for age and severity, suggesting more complex needs and potential barriers to discharge.

## Discussion

This national analysis demonstrates a persistent socioeconomic gradient in recorded neurotrauma admissions in Scotland between 2014 and 2020. Individuals living in the most deprived areas consistently accounted for the greatest share of admissions, whereas those in the least deprived areas accounted for the smallest share. As SIMD quintiles represent approximately equal population shares, these findings indicate disproportionate representation of deprived communities within neurotrauma admissions (5). This pattern is notable because it persists within a universal healthcare system, suggesting that socioeconomic inequality remains strongly associated with injury risk and hospital burden even where formal access to care is not insurance-based.

Several mechanisms may contribute to this gradient. Deprivation may increase exposure to injury through differences in occupational risk, environmental hazards, housing conditions, violence, and alcohol- or substance-related harms. It may also influence vulnerability after injury through higher comorbidity burden, frailty, poorer baseline health, and reduced access to social and practical support during recovery. Scottish service-mapping work has highlighted the complexity of traumatic brain injury pathways across the country, including variation in rehabilitation provision, step-down care, and onward community support (4). These factors may be particularly relevant in more deprived populations, where discharge planning and post-acute recovery can be more challenging.

The finding that length of stay was longer among more deprived groups is clinically and operationally important. Prolonged hospitalization may reflect greater injury complexity, multimorbidity, or social barriers to discharge, including availability of rehabilitation, community services, and appropriate social care placements. SABIN has previously highlighted delays and bottlenecks in rehabilitation and onward placement within Scottish brain injury care pathways, suggesting that inequalities may extend beyond initial admission and into recovery and reintegration (4). Although our dataset does not permit direct examination of the mechanisms underlying prolonged LOS, the finding is consistent with the possibility that deprivation shapes both injury burden and downstream care needs.

The observed increase in recorded admissions over the study period also requires careful interpretation. While a true increase in neurotrauma incidence cannot be excluded, the present data do not allow this trend to be attributed solely to changing epidemiology. Alternative explanations include changes in coding practice, case ascertainment, referral patterns, service organization, and administrative capture over time. We have therefore interpreted the temporal pattern cautiously and refer specifically to changes in recorded admissions rather than confirmed changes in underlying incidence.

The sex and age patterns observed in this study are consistent with the broader trauma literature. Males accounted for more admissions across injury severity strata, and distinct age-specific differences were seen between head and spinal injury patterns. Such findings support the need for targeted prevention strategies, including approaches directed at younger high-risk groups, working-age adults, and older people vulnerable to falls and frailty-related trauma (1, 4–8).

From a health-policy perspective, our findings support the use of area-based deprivation measures such as SIMD in planning trauma prevention, service provision, and rehabilitation pathways. The existence of marked socioeconomic gradients despite universal healthcare coverage suggests that equitable financing alone does not eliminate unequal exposure, unequal resilience, or unequal recovery environments. Addressing upstream determinants such as poverty, housing, violence, alcohol-related harm, and community rehabilitation capacity may be necessary if neurotrauma inequalities are to be reduced (7, 8).

## Limitations

This study has several limitations. First, it is based on administrative data and is therefore dependent on the accuracy and consistency of coding and case capture. Second, the observed increase in admissions over time may reflect changes in recording practice, ascertainment, referral pathways, or service configuration, rather than a true increase in underlying neurotrauma incidence. Third, SIMD is an area-based measure of relative deprivation and does not necessarily reflect the circumstances of every individual within a given area. Fourth, the dataset did not permit adjustment for several potentially important confounders or mediators, including mechanism of injury, ethnicity, rurality, comorbidity burden, alcohol or substance use, and detailed rehabilitation or functional outcome data. Finally, the observational nature of the analysis means that associations should not be interpreted as causal. The findings are best understood as describing population-level inequalities in recorded neurotrauma admissions and hospital stay within Scotland.

## Conclusion

Population-based data from Scotland demonstrate substantial and persistent socioeconomic gradients in recorded neurotrauma admissions and hospital length of stay across SIMD quintiles. Communities in the most deprived areas bear a disproportionate burden of neurotrauma admissions, while those in the least deprived areas are relatively under-represented. These inequalities persist within a universal healthcare system, highlighting neurotrauma as both a clinical and public health challenge. Future work should clarify the mechanisms linking deprivation to injury risk, service utilization, and recovery, and should evaluate prevention and rehabilitation strategies targeted at high-burden populations.

## Data Availability

The original contributions presented in the study are included in the article/supplementary material, further inquiries can be directed to the corresponding author.
